# The polymeric fluoropyrimidine CF10 overcomes limitations of 5-FU in pancreatic ductal adenocarcinoma cells through increased replication stress

**DOI:** 10.1080/15384047.2024.2421584

**Published:** 2024-11-08

**Authors:** Jennifer M. Finan, Roberto Di Niro, Soon Young Park, Kang Jin Jeong, Madeline D. Hedberg, Alexander Smith, Grace A. McCarthy, Alex O. Haber, John Muschler, Rosalie C. Sears, Gordon B. Mills, William H. Gmeiner, Jonathan R. Brody

**Affiliations:** a Department of Surgery, Oregon Health & Science University, Portland, OR, USA; b Brenden-Colson Center for Pancreatic Care, Oregon Health & Science University, Portland, OR, USA; c Knight Cancer Institute, Oregon Health & Science University, Portland, OR, USA; d Department of Cell, Development and Cancer Biology, Oregon Health and Sciences University, Portland, OR, USA; e Division of Oncological Sciences, Oregon Health & Science University, Portland, OR, USA; f The Jefferson Pancreas, Biliary and Related Cancer Center, Department of Surgery, Thomas Jefferson University, Philadelphia, PA, USA; g Department of Molecular and Medical Genetics, Oregon Health & Science University, Portland, OR, USA; h Department of Cancer Biology, Wake Forest School of Medicine, Winston-Salem, NC, USA

**Keywords:** PDAC, pancreatic cancer, replication stress, DNA damage, fluoropyrimidine

## Abstract

Pancreatic ductal adenocarcinoma (PDAC) is a lethal disease soon to become the second leading cause of cancer deaths in the US. Beside surgery, current therapies have narrow clinical benefits with systemic toxicities. FOLFIRINOX is the current standard of care, one component of which is 5- Fluorouracil (5-FU), which causes serious gastrointestinal and hematopoietic toxicities and is vulnerable to resistance mechanisms. Recently, we have developed polymeric fluoropyrimidines (F10, CF10) which unlike 5-FU, are, in principle, completely converted to the thymidylate synthase inhibitory metabolite FdUMP, without generating appreciable levels of ribonucleotides that cause systemic toxicities while displaying much stronger anti-cancer activity. Here, we confirm the potency of CF10 and investigate enhancement of its efficacy through combination with inhibitors in vitro targeting replication stress, a hallmark of PDAC cells. CF10 is 308-times more potent as a single agent than 5-FU and was effective in the nM range in primary patient derived models. Further, we find that activity of CF10, but not 5-FU, is enhanced through combination with inhibitors of ATR and Wee1 that regulate the S and G2 DNA damage checkpoints and can be reversed by addition of dNTPs indicative of CF10 acting, at least in part, through inducing replication stress. Our results indicate CF10 has the potential to supersede the established benefit of 5-FU in PDAC treatment and indicate novel combination approaches that should be validated in vivo and may be beneficial in established regimens that include 5-FU.

## Introduction

Pancreatic ductal adenocarcinoma (PDAC) is one of the most lethal forms of cancer with 5-year survival of only 11%. 5-FU has been reported to have activity, either alone or in combination with other agents, in PDAC patients since 1974.^1^ Burris *et al*. performed a landmark clinical trial that included 126 patients with advanced PDAC, setting the stage for the anti-metabolite gemcitabine (GEM) to be considered standard of care (SOC) over 5-FU. This was predominantly due to clinical benefits that included a modest increase in overall survival of 5.65 months in the GEM-treated arm vs 4.41 months in the 5-FU-treated arm.^2^ However, this narrow clinical benefit did not stop clinical investigations over the past 20 years from demonstrating that 5-FU and/or 5-FU derivatives (i.e., S1; and an oral version of 5-FU, capecitabine) have activity (~10-20% response rates) in a subset of PDAC patients.^3^

Currently, the best treatment option for patients with PDAC, besides surgery, are cytotoxic chemotherapies. Clinical studies in the metastatic setting revealed improved overall- and disease-free survival with a combination of cytotoxic agents as compared to SOC alone.^4,5^ In fact, FOLFIRINOX [FOL = Leucovorin LV Calcium (Folinic Acid), F = 5-Fluorouracil, IRIN = Irinotecan Hydrochloride, OX = Oxaliplatin], is becoming the drug regimen of choice, particularly in healthy, high-performance status patients with PDAC. Still, overall survival was improved only a few months, disease eventually progressed in almost all patients, and many experienced serious systemic toxicities (e.g. ≥ grade 3 neutropenia). Thus, new therapeutic approaches are urgently needed.

**Figure 1.**
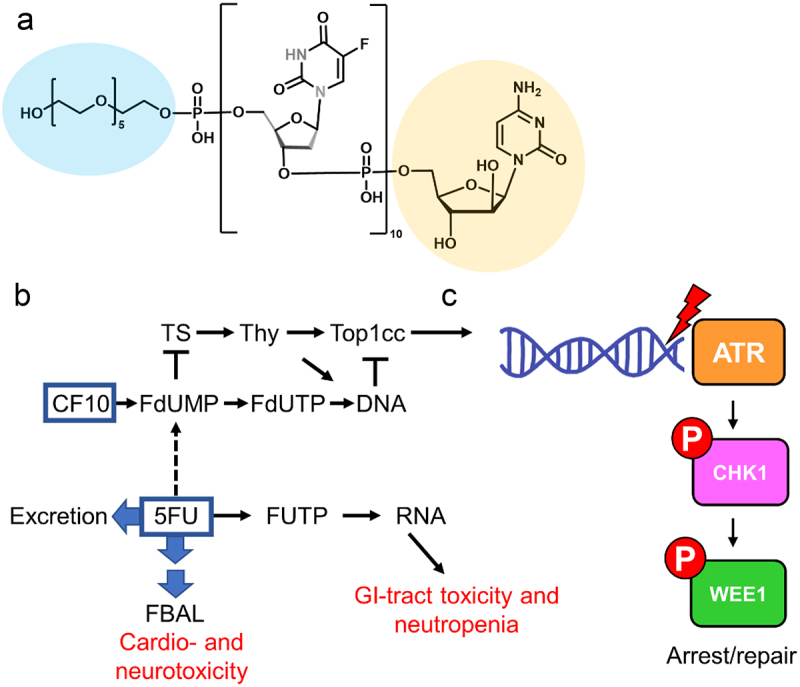
(a) Structure of CF10 with PEG6 (blue) and AraC (yellow) modifications. (b) Differential metabolism of CF10 and 5-FU resulting in increased DNA damage with CF10 treatment and vulnerability to inhibitors of DNA repair. (c) Activation of the intra-S-phase checkpoint thru the ATR/Chk1 pathway and phosphorylation of Wee1. Our studies showed that inhibition of ATR or Wee1 enhances cytotoxicity of CF10, but not 5-FU, to PDAC cells. The combination of CF10+ATRi/Wee1 inhibition may overcome 5-FU resistance for improved treatment of PDAC.

5-FU-based regimens are widely used for PDAC treatment; however, long-term survival rates remain dismal and there is an urgent need for improved therapies. To overcome the limitations of 5-FU and FOLFIRINOX for PDAC treatment, we are pioneering development of polymeric fluoropyrimidines (FPs) (e.g. F10, CF10; Figure 1). F10 is a polymer of FdUMP [reviewed in^6^] and, is completely converted to a thymidylate synthase (TS) inhibitory metabolite, without generating appreciable levels of ribonucleotides that can cause systemic toxicities.^7–9^ CF10 differs from F10 by including cytosine arabinoside (AraC) at the 3’-terminus to improve stability to enzymatic degradation and enhance poisoning of DNA topoisomerase 1,^10^ a complementary cytotoxic mechanism, not based on TS inhibition. CF10 also includes PEG6 at the 5’-terminus which facilitates cellular uptake. FP polymers cause considerably less systemic toxicities than equivalent doses of 5-FU,^11,12^ while displaying much stronger anti-cancer activity resulting in a greatly improved therapeutic index. In our recent studies, we reported that the 2^nd^ generation polymeric FP CF10 was more potent than the prototype polymer F10 and much more potent than 5-FU in multiple PDAC cell lines, including MIA PaCa-2, PANC-1, and AsPC-1.^13,14^ While 5-FU was effective at µM concentrations (GI50 range: 6.3–15.8 µM), CF10 was 185-889× more potent than 5-FU (GI50 range: 7.06–85.3 nM), greatly exceeding the 10-fold increased FP content of CF10 relative to 5-FU in each cell line. Importantly, CF10 was uniformly potent to PDAC cell lines in the nM concentration range indicating it has potential for overcoming the limited therapeutic benefit of 5-FU.

We previously established that the increased potency of CF10 relative to 5-FU correlated with increased TS inhibition and greater topoisomerase 1-DNA covalent cleavage complex (TOP1cc) formation culminating in increased DNA damage and elevated replication stress, specifically increased replication fork collapse.^14–16^ PDAC cells are subject to high levels of basal replication stress due to increased MYC expression, mutations in *KRAS* and other oncogenes and tumor suppressors that stimulate rapid, uncontrolled proliferation. Thus, inhibitors of ATR (ATRi) and Wee1 (Wee1i) kinases display promising single agent activity in PDAC cells. Further, these agents may enhance the potency of compounds that induce replication stress. In the present study, we investigate the potential of ATR and Wee1 kinase inhibition to enhance the potency of 5-FU and CF10 to determine if this combinatorial strategy can potentially overcome the limitations of current therapy options for PDAC.

## Results

### Improved potency of CF10 relative to 5-FU in PDAC cells

Our earlier reports indicated that CF10 had potential therapeutic advantages over 5-FU, a component of the typical drug regimen of FOLFIRINOX, for treatment of PDAC.^13,14^ To confirm this notion, we determined the relative potency of these agents in additional PDAC cell lines. In addition to the MIA PaCa-2, PANC-1, and AsPC-1 cell lines which we had included in previous studies, additional experiments were conducted in BxPC-3, Capan-1, Capan-2, HPAF-II, and HS 766T PDAC cell lines. Apart from BxPC-3, all of these cell lines carry activating *KRAS* mutations and with the exception of HS 766T all contain *TP53* mutations and mutation or deletion of *CDKN2A/p16*. Thus, these cell lines are representative of PDAC, which is characterized by activating *KRAS* mutations, loss or mutation of p53, and mutations or deletion of p16 (**Supplemental Table S1**).

**Figure 2.**
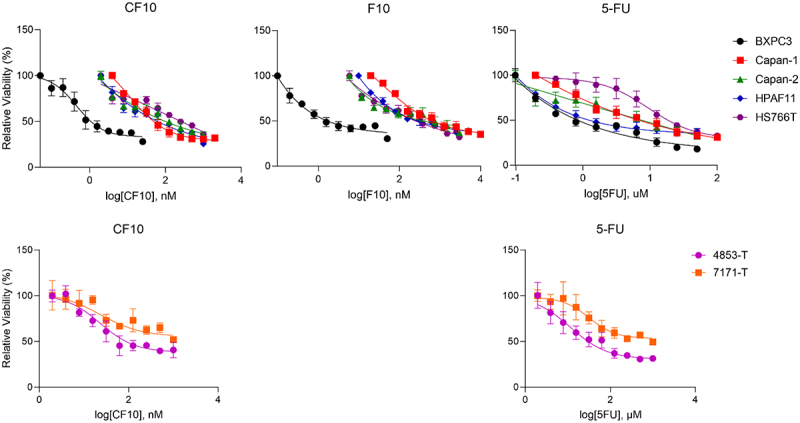
Dose-response viability curves for CF10, F10 and 5-FU in five conventional PDAC (BXPC-3, Capan1, Capan2, HPAF11, HS766T) and two primary patient-derived (7171-T and 4853-T) cell lines showing single agent efficacy. CF10 and F10 are displayed in nM while 5-FU is displayed in µm. IC50 values were calculated and reported in Table 1.

CF10 displayed strong potency in all conventional and primary patient-derived PDAC cell lines tested and was effective in the nM range (GI50 range: 3.13–333 nM). Importantly, CF10 was uniformly much more potent than 5-FU, by a 408× average factor (range 10.7 to 980×) and F10, by a 6.1× average factor (range 1.7 to 14.1×). The overall potency of 5-FU to the cell lines studied was similar to our previous studies with micromolar potency (GI50 range: 2.6–25.1 µM) (Figure 2, Table 1). Importantly, there was no significant correlation between sensitivity to CF10 and 5-FU across the lines with 5-FU-resistant lines such as HS766T, AsPC1 and PANC-1 being sensitive to CF10. Furthermore, sensitivity to CF10 was observed across lines with the different mutational events prevalent in PDAC (Table 1).

**Table 1.** Summary of IC50 values for CF10, F10, and 5-FU in select PDA conventional and patient-derived cell lines. *p* values were calculated utilizing an two-tailed Student’s *t*-test comparing CF10 to F10 or 5-FU. (†= Previously published; * = p < .05; ** = p < .01; *** = p < .001; **** = p < .0001).

IC50 (nM)	CF10	F10	5FU
BXPC3	3.13 ± 0.74	5.22 ± 22.60 (*p* = .066)	2603.00 ± 245.27 (****)
	Increased CF10 potency:	1.7x	831x
Capan-1	101.80 ± 22.60	1357.00 ± 230.70 (***)	10627.00 ± 817.90 (****)
	Increased CF10 potency:	13.3x	104x
Capan-2	131.50 ± 48.50	567.50 ± 27.10 (***)	8193.00 ± 659.60 (****)
	Increased CF10 potency:	4.3x	62.3x
HPAF-II	333.60 ± 52.00	566.30 ± 29.70 (**)	3557.00 ± 370.20 (***)
	Increased CF10 potency:	1.7x	10.7x
HS 766T	217.90 ± 38.12	480.80 ± 87.10 (**)	25093.00 ± 4382.00 (***)
	Increased CF10 potency:	2.2x	115x
AsPC-1†	85.30 ± 4.62	1203.00 ± 308.70 (**)	15820.00 ± 529.90 (****)
	Increased CF10 potency:	14.1x	185x
MIA PaCa-2†	7.06 ± 2.89	25.43 ± 4.00 (**)	6277.00 ± 426.00 (****)
	Increased CF10 potency:	3.6x	889x
Panc-1†	46.01 ± 1.19	345.10 ± 33.00 (****)	12340.00 ± 1163.00 (****)
	Increased CF10 potency:	7.5x	268x
4853-T	18.08		11480(**)
	Increased CF10 potency:		638x
7171-T	32.66		31990(**)
	Increased CF1- potency:		1000x
	Combined increased average CF10 potency:	6.1x	308x
Correlation vs 5FU	CF10 vs 5FU: R^2^, 0.03, *p* value 0.68, not significant.	F10 vs 5FU: R^2^, 0.11, *p* value 0.41, not significant.	

### CF10 induces replication stress

**Figure 3.**
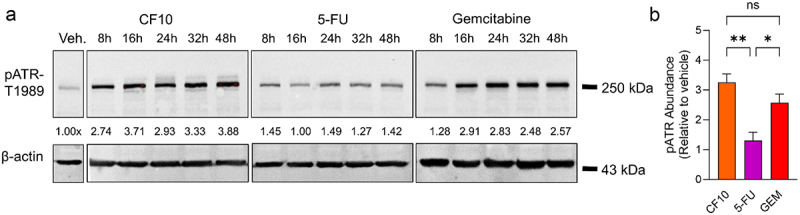
(a) Representative Western blot detecting phosphorylation of ATR at threonine 1989 in MIA PaCa-2 cells after treatment with CF10, 5-FU, or GEM. Protein was collected at 5 time points between 8 and 48 hours, with each drug administered at IC50 concentration. Intensity of pATR (top) was normalized to a loading control (β-actin, bottom) and shown as fold increase as compared to vehicle treated (Veh.) cells. (b) Plotted relative pATR expression of three biological replicates in MIA PaCa-2 cells at 48 hours of CF10 (white), 5-FU (blue), or GEM (red) treatment. *p* values were calculated using an ordinary one-way ANOVA with significance defined as *p* < .05. *, *p* < .05; **, *p* < .01; ***, *p* < .001; ns, not significant.

Our previous studies indicated that CF10 displays dual mechanistic action, targeting and inhibiting TS and DNA topoisomerase 1 (Top1).^10,11,17,18^ Importantly, we established this dual targeting mechanism is activated in multiple PDAC cell lines. In contrast, approximately 1,000-fold higher concentrations of 5-FU were required to achieve a similar extent of TS inhibition. Importantly, this is much higher than the 10-fold increase in FP content. Further, while 5-FU also induced TOP1cc, this required much higher concentrations than for CF10, and unlike CF10, 5-FU-induced TOP1cc were reversed by uridine, consistent with the induction of TOP1cc being due to effect of RNA analogs rather than DNA analogs. To gain further insight into differential effects of CF10 and 5-FU in PDAC cells, we assessed the phosphorylation of ATR, a kinase that is activated by phosphorylation in response to replication stress (Figure 3, Supplemental Figure S1).

MIA PaCa-2 cells were treated with CF10, 5-FU or GEM at their respective IC50 concentrations. Our results indicated that CF10, but not 5-FU, induces activation of ATR (Figure 3). Phosphorylation was evident at all time points and showed the highest value at the 48 hour time point, consistent with CF10 inducing DNA damage with slightly slower kinetics, which is likely a consequence of the fact that FdU needs to be released from CF10 and incorporated into DNA, a process requiring several hours. Importantly, phosphorylation of ATR induced by CF10 was significantly higher than with 5-FU, both in absolute terms and relative to IC50 values.

### ATR and Wee1 inhibition enhance CF10 potency in PDAC

**Figure 4.**
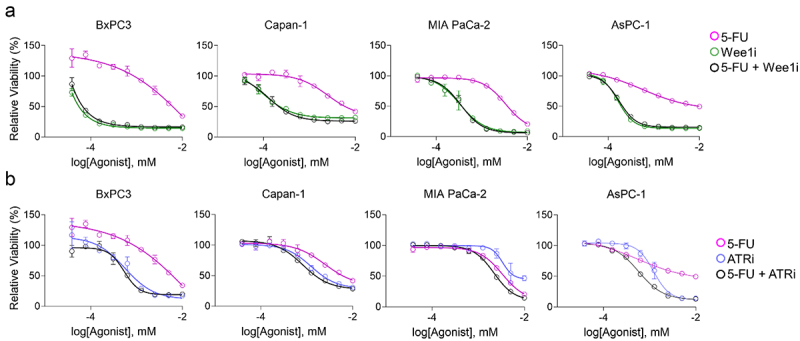
Viability effect of Wee1i or ATRi with single agent compounds in four PDAC cell lines. (a) The Wee1 inhibitor AZD1775 (adavosertib, green) showed increased single agent efficacy as compared to 5-FU (magenta), while treatment with both drugs were not additive (black). (b) ATRi (blue) showed increased single agent efficacy as compared to 5-FU (magenta), while treatment with both drugs did not show an additive effect (black).

Our previous studies demonstrated that CF10, but not 5-FU, cause replication fork collapse, and that CF10 was a potent inducer of replication stress in PDAC^13^ and other cancer cell lines.^16^ Therefore, we further investigated to what extent ATRi and Wee1i could selectively enhance the efficacy of CF10 on PDAC cells. The rationale for this inhibition is strong as these kinases induce S-phase slowing to allow cells to recover from replication stress and abrogate the G2-checkpoint that allows cells to repair double stranded break induced by replication stress. We first tested the activity of the Wee1i (AZD1775; adavosertib) and ATRi (AZD6738) as single agents in multiple PDAC cell lines (BxPC-3, CAPAN-1, MIA PaCa-2, AsPC-1) and found that each displayed significant activity in all PDAC cell lines tested. In fact, the potency of Wee1i (Figure 4a) and ATRi (Figure 4b) as single agents were similar to or better than 5-FU, an established drug in PDAC, a result consistent with PDAC cells being under high levels of inherent replication stress. Combining 5-FU with ATRi or Wee1i; however, had minimal or no enhancement and the combination displayed similar dose-response profile to the most efficient single agents. Our findings are consistent with 5-FU potency not being enhanced through inhibition of the ATR/CHK1/Wee1 signaling cascade.

**Figure 5.**
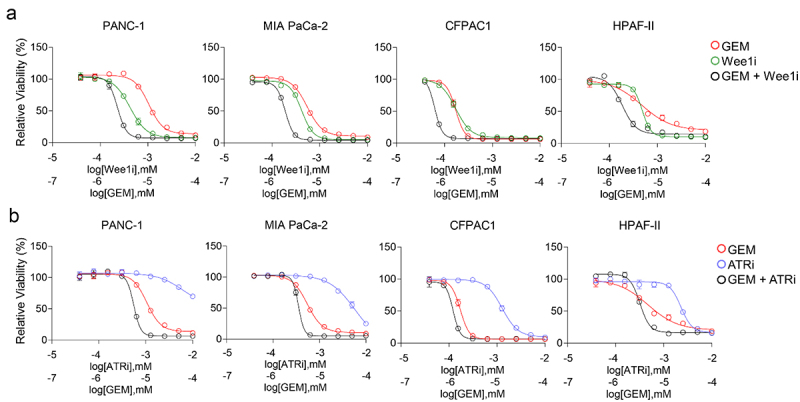
Wee1i and ATRi showed single agent compound efficacy in in four PDAC cell lines, and cytotoxicity is enhanced in combination with GEM. (a) The Wee1i (green) showed single agent efficacy comparable to or better than GEM (red), while concurrent treatment with both drugs displayed enhanced efficacy in all cell lines tested (black). (b) The ATRi (blue) showed single agent efficacy in all cell lines tested as well as enhanced cytotoxicity when administered concurrently to GEM (black).

We next evaluated GEM, a drug commonly used as frontline treatment for PDAC, for its interaction with ATRi and Wee1i in PDAC cells. GEM mediates its activity through multiple DNA-directed pathways including ribonucleotide reductase (RNR) leading to deoxynucleotide depletion, inhibition of DNA polymerase extension, and poisoning of DNA Top1.^10,17^ Consistent with GEM inducing increased levels of replication stress in PDAC cells, we found that combining it with ATRi (AZD6738) or Wee1i (AZD1775) resulted in increased cytotoxicity relative to single agents in four different pancreatic cancer cell lines (PANC-1, MIA PaCa-2, CFPAC1, HPAF-II) (Figure 5).

**Figure 6.**
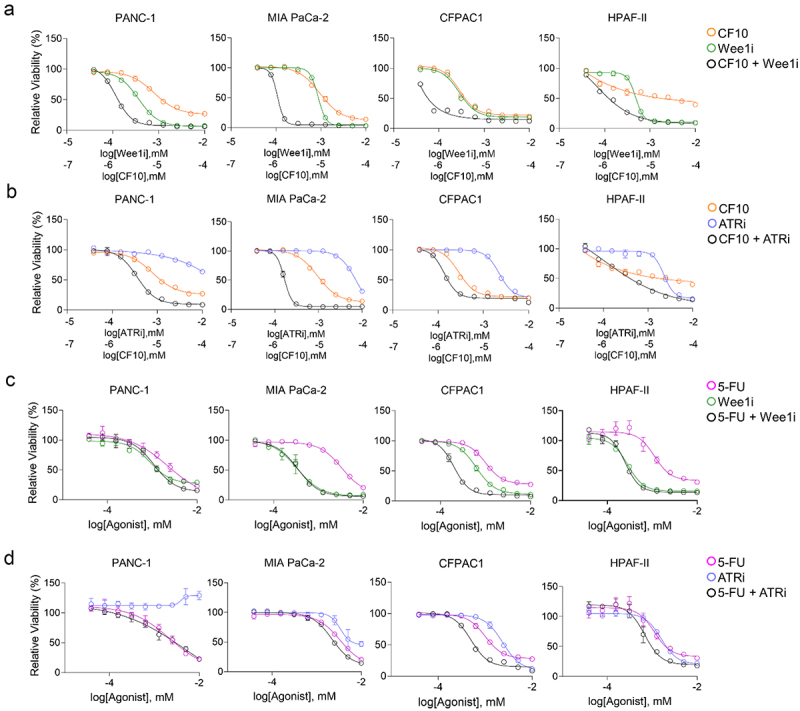
CF10, but not 5-FU, is enhanced by Wee1i and ATRi in four PDAC cell lines. (a, b) the Wee1i (green) and the ATRi (blue) showed enhanced cytotoxicity in all tested cell lines when administered concurrently to CF10 (black, single agent orange). (c, d) Conversely, enhanced effect is not seen in three out of four cell lines, and only modestly in the CFPAC1 cell line, when the Wee1i (green) and ATRi (blue) were administered either concurrently (black) to 5-FU (single agent, magenta).

CF10 displays some mechanistic similarities to GEM, including formation of TOP1cc^10,15–17^ and was therefore also evaluated in these PDAC cells. As a single agent, CF10 displayed similar potency to that of GEM, consistent with CF10 having potential for PDAC treatment (Figure 2). Importantly, CF10 activity was enhanced through combination with ATRi and Wee1i (Figure 6a,b). We observed that this effect was more pronounced when drugs were administered at the same time, rather than sequentially (data not shown). The specificity of this result was confirmed by testing 5-FU in combination with ATRi and Wee1i in these same cell lines, demonstrating that there was no interaction between 5-FU and ATRi or Wee1i (Figure 6c,d).

### dNTPs rescue viability of cells treated with CF10

**Figure 7.**
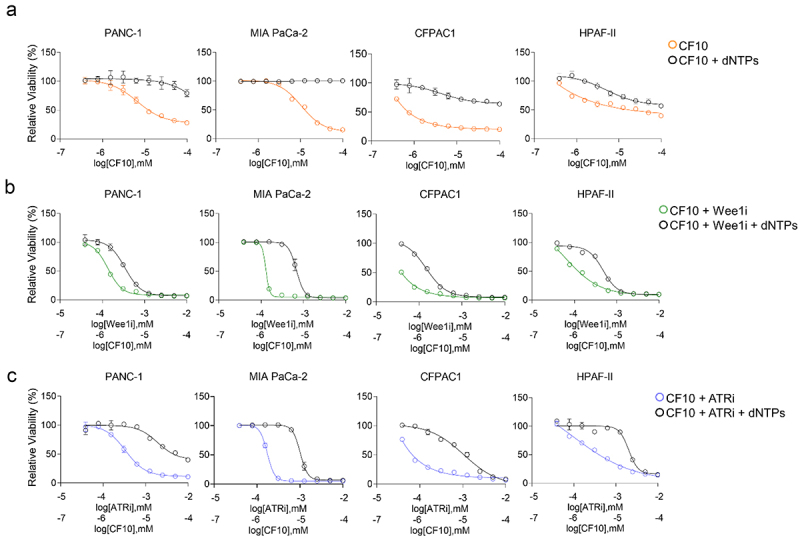
Adding of deoxynucleotides rescues viability in cells treated with CF10 or CF10+Wee1i/ATRi. (a) Four PDAC cell lines were treated with CF10 (orange) or CF10 and 10 µm dNTPs (black). (b) The same cell lines were tested with CF10 + Wee1i (green) or CF10 + Wee1i and 10 µm dNTPs (black). (c) The same cell lines were tested with CF10 + ATRi (blue) or CF10 + ATRi and 10 µm dNTPs (black).

Since nucleotide deprivation is a potential cause for replication stress, we investigated whether addition of dNTPs could rescue the cell viability of lines treated with CF10 and/or ATRi/Wee1i. We found that in all cell lines tested, viability was noticeably rescued when 10 µM dNTPs were added to cells treated with either CF10 or with a combination of CF10 and ATRi/Wee1i (Figure 7).

Importantly, this effect was specific to CF10, as cells treated with ATR or Wee1 inhibitors alone did not show viability rescue in presence of dNTPs, and in one case, this induced even higher sensitivity (**Supplemental Figure S2**). Taken together, our results thus indicate that CF10 has potential to be useful for PDAC treatment and that novel combination strategies to enhance its efficacy through increased replication stress by inhibition of ATR and Wee1 may be highly effective.

## Discussion

Our studies represent the first attempt to exploit the increased DNA-directed activity of the polymeric FP CF10 relative to 5-FU to develop novel and highly effective combination treatments for PDAC. 5-FU remains an important component of therapeutic regimens to treat PDAC, notably as an integral component of FOLFIRINOX, a preferred front-line treatment in high-performance PDAC patients. Our previous studies demonstrated CF10 is more potent than 5-FU in multiple PDAC cell lines, beyond the 10-fold increase in FP content. In the present study, we extended these findings to multiple additional conventional and primary patient-derived PDAC cell lines that collectively display mutational profiles characteristic of PDAC, consistent with the increased potency of CF10 relative to 5-FU being generally applicable. Interestingly, we found that the one *BRAF* mutant cell line, BxPC-3, was more sensitive to CF10 when compared to KRAS mutant cell lines. These data are consistent with findings that ERK feedback is impaired in *BRAF* but not *KRAS* mutant cells, suggesting they may be more vulnerable to replications stress.^19,20^ Further work is warranted to investigate this association.

In our previous studies, we also demonstrated that the improved potency of CF10 relative to 5-FU correlated with increased TS inhibition, elevated TOP1cc formation, and replication fork slowing and collapse, all indicative of heightened replication stress.^15,16^ PDAC cells are under relatively higher inherent levels of replication stress relative to nonmalignant cells and less aggressive malignancies.^21,22^ This has resulted in evaluation of inhibitors of the ATR/CHK1/WEE1 cascade as potential therapeutic options for PDAC. In the present studies we determined that the increased potency of CF10 relative to 5-FU was associated with increased activation of ATR, a kinase activated upon replication fork collapse. Further, we demonstrated that treatment with CF10 resulted in significantly higher ATR activation than for 5-FU, highlighting CF10’s improved potency.

CF10 strongly activated ATR and in this regard displayed greater similarities to GEM, a nucleoside analog widely used in front-line treatment of PDAC, than to 5-FU. Intriguingly, both CF10 and GEM are implicated in suppressing dNTP levels but through complementary mechanisms – GEM through inhibiting RNR and CF10 through inhibiting TS. Intriguingly, both CF10 and GEM poison Top1 after incorporation of the corresponding triphosphate into DNA, although CF10 also shows differences in Tdp1-mediated TOP1cc repair.^10,15,23,24^ Thus, CF10 and GEM are both capable of causing replication stress via two distinct processes, nucleotide pool depletion^25^ and poisoning of Top1,^26^ but target these processes via different mechanisms. The induction of replication stress by multiple processes may be particularly beneficial for therapy of malignant cells, such as PDAC, that are under relatively high levels of inherent replication stress. It also suggests that PDAC cells may be particularly reliant upon the ATR/Chk1/Wee1 kinase cascade for survival upon treatment. Indeed, our studies showed that both CF10 and GEM displayed improved potency in combination with both ATRi and Wee1i. In contrast, 5-FU activity was not improved with these combinations.

We further investigated the contribution of dNTP depletion for CF10 cytotoxicity. While at least partial rescue was detected in all cell lines tested, the relative efficiency was highly variable among the different cell lines tested consistent with the variable contribution of Top1 poisoning among the cell lines tested. In all cases, the combination of CF10 with either ATRi or Wee1i was relatively more resilient to reversal with exogenous dNTPs than for treatment with CF10 alone. These findings suggest that addition of ATRi or Wee1i in combination with CF10 advances PDAC cells to a state of irreversible DNA damage resulting from unresolved replication stress rendering them impervious to the availability of increased dNTP pools. These findings may have therapeutic consequences signifying the potential of CF10 plus ATRi and Wee1i for more complete tumor eradication.

## Conclusions

Overall, our results indicate that CF10 offers novel potential as the FP component of combination chemotherapy regimens to treat PDAC. While FOLFIRINOX is emerging as a preferred SOC therapy for PDAC patients, its use is restricted by several factors associated with limitations of 5-FU. The anti-cancer activity of this regimen is likely sub-optimal because 5-FU is very inefficiently metabolized to the target metabolite, FdUMP,^27–29^ limiting DNA-directed activities including generation of replication stress. CF10 as a single agent effectively targets two of the enzymatic targets of FOLFIRINOX (TS and Top1, targeted by 5-FU and irinotecan, respectively); therefore, the use of CF10 permits expanding the combinatorial arsenal to include agents specifically directed at replication stress, such as ATRi and Wee1i. While ATRi and Wee1i have shown toxicities in clinical trials, our previous studies with CF10 demonstrate very low systemic toxicity. Thus, CF10 plus ATRi/Wee1i warrants further investigation in more sophisticated pre-clinical models for clinical development.

In summary, we have expanded the range of PDAC cell models demonstrated to show strong sensitivity to CF10 and a substantially improved potency for CF10 relative to 5-FU, well above the increase in FP content of the drugs. Our studies highlight the increased activation of ATR, indicative of replication stress, induced by CF10 relative to 5-FU. While PDAC cells are under relatively high levels of replication stress and are sensitive to ATRi and Wee1i, these cells are especially sensitive to the combination of these agents with CF10. Further, the use of CF10 in these combinations reduces the reversal of cytotoxicity by exogenous dNTPs indicating causation of irreversible DNA damage, potentially through induction of mitotic catastrophe. Our work points to the possibility of CF10 and CF10 combinations with inhibitors of the ATR/Chk1/Wee1 signaling cascade as being therapeutically viable alternatives to current front-line regimens for PDAC including FOLFIRINOX and GEM. More work is required to enhance and scale up production of 5-FU to obtain enough drug for *in vivo* studies. Ultimately, future studies are needed to test the potential of these therapeutic approaches in mouse models of PDAC.

## Experimental procedures

### Cell line culture conditions

All cell lines were purchased from American Type Culture Collection (ATCC) and are reported in Supplemental Table 1 along with their mutational content relevant to PDAC. Cells were authenticated by short tandem repeat profiling at least twice per year and confirmed negative for mycoplasma contamination at least once per month. Cells were cultured according to ATCC specifications with the addition of prophylactic plasmocin (InvivoGen). For all experiments cell line passage number was kept below 20. Primary patient-derived PDAC cell lines (7171-T and 4853-T) were generated at Oregon Health & Science University. Cells were cultured in CRC media (1 part Gibco F-12 Medium: 3 parts Gibco DMEM, 5% FBS, 0.4 μg/ml hydrocortisone, 5 μg/ml human insulin, 8.4 ng/ml cholera toxin, 10 ng/ml EGF, 24 μg/ml adenine, 1X Primocin, 10 µM Rho kinase inhibitor). For all experiments cell line passage number was kept below 20.

### Chemical compounds

F10 was synthesized and characterized as previously described.^7,8^ F10 and CF10 were synthesized and provided by Dr. William Gmeiner^13,14^ and both were prepared in saline. The Wee1 inhibitor AZD1775 (adavosertib) and the ATR inhibitor AZD6738 were obtained from Selleck Chemicals.

### Cell viability assays

1000 cells/well were plated in 96-well plates. Drug treatment of cells was performed in triplicate, with dosage starting the day after plating. Cell viability was assessed after 6 days using the PrestoBlue reagent (ThermoFisher) following the manufacturer’s recommendation. For sequential treatments, cells were treated for three days with each drug before assessment of cell viability. Rescue experiments were performed by adding deoxynucleotides (10 µM) at the same time as the other drug(s). Graphs were plotted using GraphPad Prism software.

### Western blotting

Cells were treated with compounds as indicated in figures and figure legends, and samples were collected at 8, 16, 24, 32 and 48 hours. Cells lysis was performed with the RIPA lysis buffer system (Santa Cruz Biotechnology) and soluble protein content quantified by BCA protein assay (Pierce). Proteins were separated with a 10% SDS-PAGE and transferred onto a PVDF membrane (BioRad) following standard procedures. Membranes were blocked with Li-COR TBS Intercept blocking buffer (Li-COR) for 30 minutes at RT, incubated with primary antibodies overnight at 4°C and with secondary antibodies for 1 h at room temperature. 3× washes in PBS added with 0.1% Tween-20 were performed in between antibodies and before imaging. Primary antibodies were as follows: pATR-T1989 (#30632, Cell Signaling); β-actin (#AM4302, ThermoFisher Scientific). Secondary antibodies were as follows: goat-anti-mouse AlexaFluorPlus-555 (#A32727); goat anti-rabbit AlexaFluorPlus-800 (#A32735). Imaging was performed on an iBright-1500 (ThermoFisher Scientific). Bands were quantified using Image J and graphs were plotted using GraphPad Prism software. Plotted experiments were performed in biological triplicate.

### Statistical analysis

Two tailed Student’s *t*-test were performed using GraphPad Prism software (San Diego, CA) for all analyses unless otherwise indicated. Results are expressed as mean ± standard error of mean. For correlation of treatments among all cell lines, statistical analysis was calculated using Pearson’s correlation.

## Supplementary Material

Supporting information.docx

## Data Availability

The data supporting the conclusions of this manuscript are available from the corresponding author upon reasonable request. All data supporting the findings of this study are included within the article and its supplemental data.

## References

[1] Kovach JS, Moertel CG, Schutt AJ, Hahn RG, Reitemeier RJ. Proceedings: a controlled study of combined 1,3-bis-(2-chloroethyl)-1-nitrosourea and 5-fluorouracil therapy for advanced gastric and pancreatic cancer. Cancer. 1974;33:563–10. doi:10.1002/1097-0142(197402)33:2<563:aid-cncr2820330235>3.0.co;2-k. 4812773

[2] Burris HA 3rd, Moore MJ, Andersen J, Green MR, Rothenberg ML, Modiano MR, Cripps MC, Portenoy RK, Storniolo AM, Tarassoff P, et al. Improvements in survival and clinical benefit with gemcitabine as first-line therapy for patients with advanced pancreas cancer: a randomized trial. J Clin Oncol. 1997;15(6):2403–2413. doi:10.1200/JCO.1997.15.6.2403. 9196156

[3] Lawrence B, andFindlay M. Systemic therapy for metastatic pancreatic adenocarcinoma. Ther Adv Med Oncol. 2010;2(2):85–106. doi:10.1177/1758834009357188. 21789129 PMC3126009

[4] Conroy T, Desseigne F, Ychou M, Bouche O, Guimbaud R, Becouarn Y, Adenis A, Raoul J-L, Gourgou-Bourgade S, de la Fouchardière C, et al. FOLFIRINOX versus gemcitabine for metastatic pancreatic cancer. N Engl J Med. 2011;364(19):1817–1825. doi:10.1056/NEJMoa1011923. 21561347

[5] Von Hoff DD, Ramanathan RK, Borad MJ, Laheru DA, Smith LS, Wood TE, Korn RL, Desai N, Trieu V, Iglesias JL, et al. Gemcitabine plus nab-paclitaxel is an active regimen in patients with advanced pancreatic cancer: a phase I/II trial. J Clin Oncol. 2011;29(34):4548–4554. doi:10.1200/JCO.2011.36.5742. 21969517 PMC3565012

[6] Gmeiner WH, Debinski W, Milligan C, Caudell D, andPardee TS. The applications of the novel polymeric fluoropyrimidine F10 in cancer treatment: current evidence. Future Oncol. 2016;12(17):2009–2020. doi:10.2217/fon-2016-0091. 27279153 PMC4992963

[7] Gmeiner WH, Trump E, andWei C. Enhanced dna-directed effects of FdUMP[10] compared to 5FU. Nucleosides, Nucleotides & Nucleic Acids. 2004;23(1–2):401–410. doi:10.1081/ncn-120028336. 15043163

[8] Liu J, Skradis A, Kolar C, Kolath J, Anderson J, Lawson T, Talmadge J, Gmeiner WH. Increased cytotoxicity and decreased in vivo toxicity of FdUMP[10] relative to 5-FU. Nucleosides Nucleotides. 1999;18(8):1789–1802. http://www.ncbi.nlm.nih.gov/pubmed/10478484. 10478484 10.1080/07328319908044843

[9] Liu J, Kolar C, Lawson TA, andGmeiner WH. Targeted drug delivery to chemoresistant cells: folic acid derivatization of FdUMP[10] enhances cytotoxicity toward 5-fu-resistant human colorectal tumor cells. J Org Chem. 2001;66(17):5655–5663. doi:10.1021/jo005757n. 11511236

[10] Gmeiner WH. Entrapment of DNA topoisomerase-dna complexes by nucleotide/nucleoside analogs. Cancer Drug Resist. 2019;2:994–1001. doi:10.20517/cdr.2019.95. 31930190 PMC6953902

[11] Pardee TS, Gomes E, Jennings-Gee J, Caudell D, andGmeiner WH. Unique dual targeting of thymidylate synthase and topoisomerase1 by FdUMP[10] results in high efficacy against AML and low toxicity. Blood. 2012;119(15):3561–3570. doi:10.1182/blood-2011-06-362442[pii]. 22362039 PMC3325043

[12] Pardee TS, Stadelman K, Jennings-Gee J, Caudell DL, andGmeiner WH. The poison oligonucleotide F10 is highly effective against acute lymphoblastic leukemia while sparing normal hematopoietic cells. Oncotarget. 2014;5(12):4170–4179. doi:10.18632/oncotarget.1937. 24961587 PMC4147314

[13] Haber AO, Jain A, Mani C, Nevler A, Agostini LC, Golan T, Palle K, Yeo CJ, Gmeiner WH, Brody JR. AraC-FdUMP[10] is a next-generation fluoropyrimidine with potent antitumor activity in PDAC and Synergy with PARG inhibition. Mol Cancer Res. 2021;19(4):565–572. doi:10.1158/1541-7786.MCR-20-0985. 33593942 PMC9013283

[14] Gmeiner WH, Dominijanni A, Haber AO, Ghiraldeli LP, Caudell DL, D’Agostino R Jr., Pasche BC, Smith TL, Deng Z, Kiren S, et al. Improved antitumor activity of the fluoropyrimidine polymer CF10 in preclinical colorectal cancer models through distinct mechanistic and pharmacologic properties. Mol Cancer Ther. 2021;20(3):553–563. doi:10.1158/1535-7163.MCT-20-0516. 33361273 PMC8201368

[15] Jennings-Gee J, Pardee TS, andGmeiner WH. Replication-dependent irreversible topoisomerase 1 poisoning is responsible for FdUMP[10] anti-leukemic activity. Exp Hematol. 2013;41(2):180–188.e184. doi:10.1016/j.exphem.2012.10.007. 23085462 PMC3660094

[16] Mani C, Pai S, Papke CM, Palle K, andGmeiner WH. Thymineless death by the fluoropyrimidine polymer F10 involves replication fork collapse and is enhanced by Chk1 inhibition. Neoplasia. 2018;20(12):1236–1245. doi:10.1016/j.neo.2018.10.006. 30439567 PMC6232621

[17] Gmeiner WH, Andvan Waardenburg R. Targeting DNA topoisomerases: past & future. Cancer Drug Resist. 2021;4:758–761. doi:10.20517/cdr.2021.65. 34532656 PMC8442622

[18] Liao ZY, Sordet O, Zhang HL, Kohlhagen G, Antony S, Gmeiner WH, Pommier Y. A novel polypyrimidine antitumor agent FdUMP[10] induces thymineless death with topoisomerase I-DNA complexes. Cancer Res. 2005;65(11):4844–4851. doi:10.1158/0008-5472.CAN-04-1302. 15930305

[19] Heidorn SJ, Milagre C, Whittaker S, Nourry A, Niculescu-Duvas I, Dhomen N, Hussain J, Reis-Filho JS, Springer CJ, Pritchard C, et al. Kinase-dead BRAF and oncogenic RAS cooperate to drive tumor progression through CRAF. Cell. 2010;140(2):209–221. doi:10.1016/j.cell.2009.12.040. 20141835 PMC2872605

[20] Hatzivassiliou G, Song K, Yen I, Brandhuber BJ, Anderson DJ, Alvarado R, Ludlam MJC, Stokoe D, Gloor SL, Vigers G, et al. RAF inhibitors prime wild-type RAF to activate the MAPK pathway and enhance growth. Nature. 2010;464(7287):431–435. doi:10.1038/nature08833. 20130576

[21] Ubhi T, andBrown GW. Exploiting DNA replication stress for cancer treatment. Cancer Res. 2019;79(8):1730–1739. doi:10.1158/0008-5472.CAN-18-3631. 30967400

[22] Puigvert JC, Sanjiv K, andHelleday T. Targeting DNA repair, DNA metabolism and replication stress as anti-cancer strategies. FEBS J. 2016;283(2):232–245. doi:10.1111/febs.13574. 26507796

[23] Gmeiner WH, Gearhart PJ, Pommier Y, andNakamura J. F10 cytotoxicity via topoisomerase I cleavage complex repair consistent with a unique mechanism for thymineless death. Future Oncol. 2016;12(19):2183–2188. doi:10.2217/fon-2016-0127. 27333295 PMC5551924

[24] Dominijanni A, andGmeiner WH. Improved potency of F10 relative to 5-fluorouracil in colorectal cancer cells with p53 mutations. Cancer Drug Resist. 2018;1(1):48–58. doi:10.20517/cdr.2018.01. 30613833 PMC6320232

[25] Petermann E, Orta ML, Issaeva N, Schultz N, andHelleday T. Hydroxyurea-stalled replication forks become progressively inactivated and require two different RAD51-mediated pathways for restart and repair. Mol Cell. 2010;37(4):492–502. doi:10.1016/j.molcel.2010.01.021. 20188668 PMC2958316

[26] Regairaz M, Zhang YW, Fu H, Agama KK, Tata N, Agrawal S, Aladjem MI, Pommier Y. Mus81-mediated DNA cleavage resolves replication forks stalled by topoisomerase I–DNA complexes. J Cell Biol. 2011;195(5):739–749. doi:10.1083/jcb.201104003. 22123861 PMC3257568

[27] Longley DB, Harkin DP, andJohnston PG. 5-fluorouracil: mechanisms of action and clinical strategies. Nat Rev Cancer. 2003;3(5):330–338. doi:10.1038/nrc1074. 12724731

[28] Gmeiner WH. Novel chemical strategies for thymidylate synthase inhibition. Curr Med Chem. 2005;12(2):191–202. http://www.ncbi.nlm.nih.gov/pubmed/15638735. 10.2174/0929867053363432. 15638735

[29] Wilson PM, Danenberg PV, Johnston PG, Lenz HJ, andLadner RD. Standing the test of time: targeting thymidylate biosynthesis in cancer therapy. Nat Rev Clin Oncol. 2014;11(5):282–298. doi:10.1038/nrclinonc.2014.51. 24732946

