# Supplementary material for: The polymeric fluoropyrimidine CF10 overcomes limitations of 5-FU in pancreatic ductal adenocarcinoma cells through increased replication stress
Source: Cancer Biol Ther. 2024 Nov 8;25(1):2421584. doi: 10.1080/15384047.2024.2421584 (PMC11552260; doi:10.1080/15384047.2024.2421584)
Supplement: Supporting information.docx [file KCBT_A_2421584_SM5895.docx]

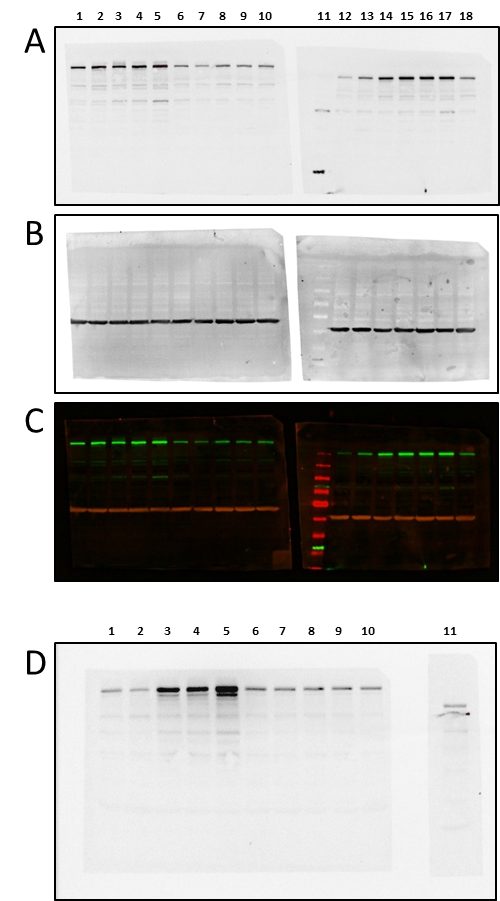


**Supplemental Figure 1.** A-C, original uncropped western blots reported in Figure 3A. Probes: A, pATR; B, β-actin; C, overlay. 1-5, CF10. 6-10, 5-FU. 11, MW. 12, non-treated. 13-17, Gemcitabine. 18, internal control. D, original uncropped blotting reported in Figure 3C. Probe: pATR. 1-5, CF10. 6-10, 5-FU. 11, non-treated.


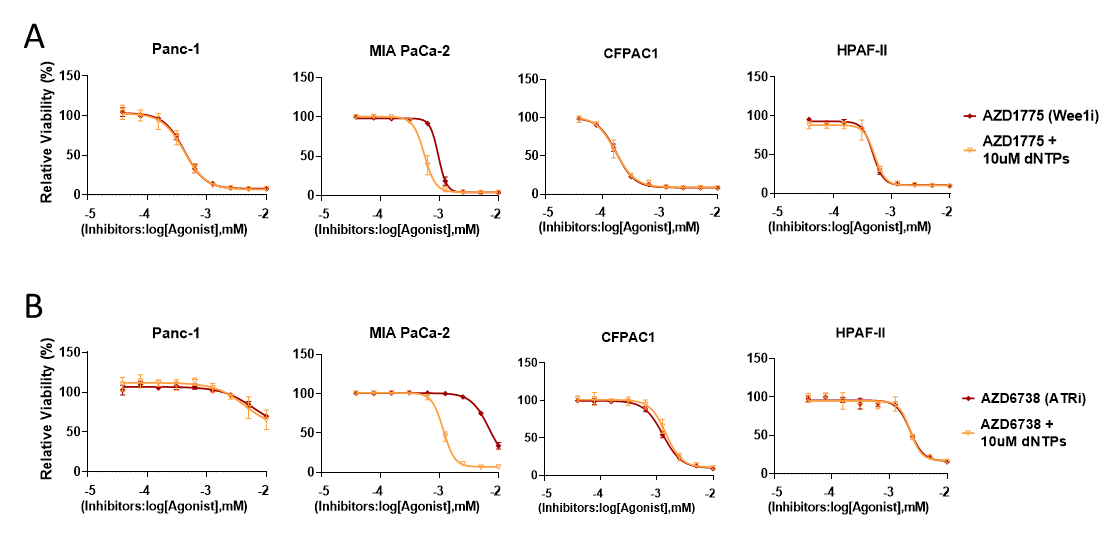


**Supplemental Figure 2.** Adding of deoxynucleotides does not rescue viability in cells treated with Wee1i or ATRi as single agents. (**A**) The same four PDA cell lines shown in Figure 7 were tested with AZD1775 (Wee1i) alone (dark red curve) or with AZD1775 + 10uM dNTPs (orange curve). (**B**) The same four PDA cell lines as above were tested with AZD6738 (ATRi) alone (dark red curve) or with AZD6738 + 10uM dNTPs (orange curve).

| Mutational Status | *KRAS* | *TP53* | *CDKN2A/p16* | *SMAD4* |
| --- | --- | --- | --- | --- |
| BXPC3 | WT | Y220C | Homozygous deletion | Homozygous deletion |
| Capan-1 | G12V | A159V | Homozygous deletion | S343STOP |
| Capan-2 | G12V | T125 SpliceVar | L78H H93P | WT |
| HPAF-II | G12D | P151S | R29A | WT |
| HS 766T | Q61H | WT | WT | Homozygous deletion |
| CFPAC1 | G12V | C242R | WT | Homozygous deletion |
| AsPC-1† | G12D | C135A | L78H H93P | R100T |
| MIA PaCa-2† | G12C | R248W | Homozygous deletion | WT |
| Panc-1† | G12D | R273H | Homozygous deletion | WT |
| 4853-T | G12V^*^ | R175H^*^ | WT | WT |
| 7171-T | G12V^*^ | M237I^*^ | WT | WT |

**Supplemental Table 1**. Summary of Mutational Status of Select PDA Cell Lines (†= Previously published)(*=Parent tumor mutation).
